# The Processing Stages for Social Interaction Recognition

**DOI:** 10.1162/OPMI.a.378

**Published:** 2026-07-17

**Authors:** Manuel Mello, Jérémy Decroix, Céline Spriet, Liuba Papeo

**Affiliations:** Institut des Sciences Cognitives—Marc Jeannerod, UMR5229, Centre National de la Recherche Scientifique (CNRS) and Université Claude Bernard Lyon 1, Bron, France

**Keywords:** social perception, scene perception, spatial cognition, action understanding, relations, event semantics

## Abstract

Understanding a social interaction requires—at least—the recognition of social agents (i.e., people), their individual actions, and the relationship between them. In what sequence do these processing steps occur? In three behavioral experiments (*n* = 184), we presented visual scenes showing bodies (or sometimes chairs), in spatial relations that implied interaction (face-to-face) or not (back-to-back), and in postures that suggested different types of interactions (i.e., ‘fighting’ or ‘dancing’). We presented these scenes for different time-durations (17, 33, 50, 67, 167 ms) followed by masking, as a way to manipulate the available processing time. In three different tasks, participants had to report the object category (body/chair), the spatial relation (facing/non-facing) or the interaction category (fighting/dancing). Categorization of people and their spatial relation yielded comparable performance at every stimulus duration, which was consistently more accurate than performance in interaction categorization. This implies that it takes no longer to access the objects’ category (bodies *vs.* chairs) than to access the relation between objects (facing *vs.* non-facing); whereas substantially longer processing time is required in order to categorize social interactions with comparable accuracy. The parallel between categorization of objects and spatial relations suggests that relations between objects are extracted as rapidly as categorical object information, providing the building blocks for social interaction recognition. These findings impose important constraints on future models of social interaction recognition.

## INTRODUCTION

Two main functions of visual perception are object location, the awareness that an object is present at a given location in the visual field, and object recognition, the assignment of an object to a category. Although spatially and functionally segregated (Goodale & Milner, 1992), the two processes are tightly linked to each other, and both are accomplished with similar delay, within a few hundreds of milliseconds (Bracci & Op de Beeck, 2023; Graumann et al., 2022; Grill-Spector & Kanwisher, 2005; Kaiser et al., 2019; see also de la Rosa et al., 2011).

But objects rarely occur in isolation. Thus, beyond detecting and recognizing an object in its absolute location (e.g., right, left, top or bottom of the visual field), individuals have to locate the object with respect to the surrounding objects that form a visual scene. Relations between objects are important to understand a scene, and to interact appropriately with the environment (Kaiser et al., 2019; Võ, 2021; Võ et al., 2019). For example, one would not grab a chair to sit down, if another is already sitting *on* that chair; or, one would not drink from a glass, if a fish is *in* that glass. Behavioral and neuroimaging evidence suggests that relations between objects are rapidly extracted from the visual input, via automatic perceptual mechanisms (Hafri & Firestone, 2021; Hafri & Papeo, 2025), and affect how single objects in a scene are processed, such that sets of objects in a meaningful, familiar configuration are detected and recognized better that the same objects in unfamiliar configurations (Kaiser et al., 2019; Papeo, 2020).

Spatial relations between objects are also important to understand social interactions, that is, events in which (at least) one agent acts to affect the physical or psychological state of (at least) another agent. Behavioral and neuroimaging evidence suggests that socially-relevant relations between people, such as whether two are mutually accessible to one another (e.g., facing) or not, are extracted in visual perception yielding visual discrimination of interacting *vs.* non-interacting people (Abassi & Papeo, 2020, 2022, 2024; Fu et al., 2024; Papeo & Abassi, 2019; Papeo et al., 2017, 2019; Vestner et al., 2019; Xu et al., 2023). Moreover, upon brief stimulus presentation (as brief as 33 ms), individuals can extract other relational cues that define the roles of individuals (i.e., agent or patient; Hafri et al., 2018; Vettori et al., 2025), and the type of interaction (Dobel et al., 2007, 2011; Hafri et al., 2013).

These research findings have motivated the hypothesis that, when we *see* an object, we also *see* the meaningful relations of that object with the surrounding objects (Hafri & Firestone, 2021; Papeo, 2020). On this view, the visual processing of scenes, such as a social interaction, involves the recognition of the entities in the scenes (e.g., two people), as well as of specific visuo-spatial cues that define whether and how those entities are connected. This view suggests that processing relationships occurs on a time scale similar to that of object recognition.

Here we asked exactly this: How does ‘relation perception’ relate to object recognition? And how does it relate to social interaction recognition? Thus, we focused on three stages involved in understanding basic social interactions: recognition of social agents, relation perception, and recognition of social interactions. Adapting a behavioral paradigm previously used to study visual object recognition (de la Rosa et al., 2011; Grill-Spector & Kanwisher, 2005), we presented visual scenes featuring two people, during three different tasks. These tasks tested, respectively: (i) recognition of human bodies *vs.* chairs used as fillers (object categorization), (ii) recognition of socially-relevant spatial relations: facing *vs.* non-facing (relation categorization), and (iii) recognition of fighting *vs.* dancing interactions (interaction categorization). In each task, stimuli were presented for different durations, followed by a masking stimulus to truncate the stimulus processing at different points in time (Breitmeyer & Ogmen, 2000; Enns & Di Lollo, 2000). We reasoned that, if a task was allowed sufficient processing time (based on stimulus duration before masking), task performance should be accurate and significantly better than a task for which the stimulus duration was too short to allow stimulus processing to occur and be completed successfully. With this approach, we compared the processing time required for the three tasks, in order to temporally characterize relation perception relative to object and action categorization. In doing so, the present study contributes to building a model that situates relation perception in the processing stream for understanding visual social interactions.

## EXPERIMENT 1

### Materials and Methods

#### Participants.

A total of 63 participants were recruited and tested online via Testable Minds, an online platform gathering participants worldwide with an advanced verification system ensuring testing reliability (Rezlescu et al., 2020). The inclusion criteria for the current study were: age between 18 and 40 years, English as native language, geographical location in Europe or North America, no report of psychiatric or neurological conditions, and normal or corrected-to-normal vision. Participants were randomly assigned to one of three groups, one for each of three experimental tasks (group 1: *n* = 20, *M*_age_ = 27, *SD*_age_ = 5, 9 females; group 2: *n* = 21, *M*_age_ = 27, *SD*_age_ = 5, 6 females; group 3: *n* = 22, *M*_age_ = 27, *SD*_age_ = 5, 8 females). The experiment was carried out in accordance with the Declaration of Helsinki (World Medical Association, 2013), and approved by the local ethics committee. Participants gave informed consent and were paid 1$ for their participation.

The sample size (*N* = 63) matched the sample size in Grill-Spector and Kanwisher (2005), and was larger than the sample required to obtain a comparable effect size (*N* = 5, for *d* > 2 with *α* = 0.05 and *β* = 0.8, two-tailed) or a generic medium effect size (*N* = 35, for *d* = 0.5 with *α* = 0.05 and *β* = 0.8, two-tailed; G*power 3.1 software; Faul et al., 2009).

#### Stimuli.

**Figure 1.**
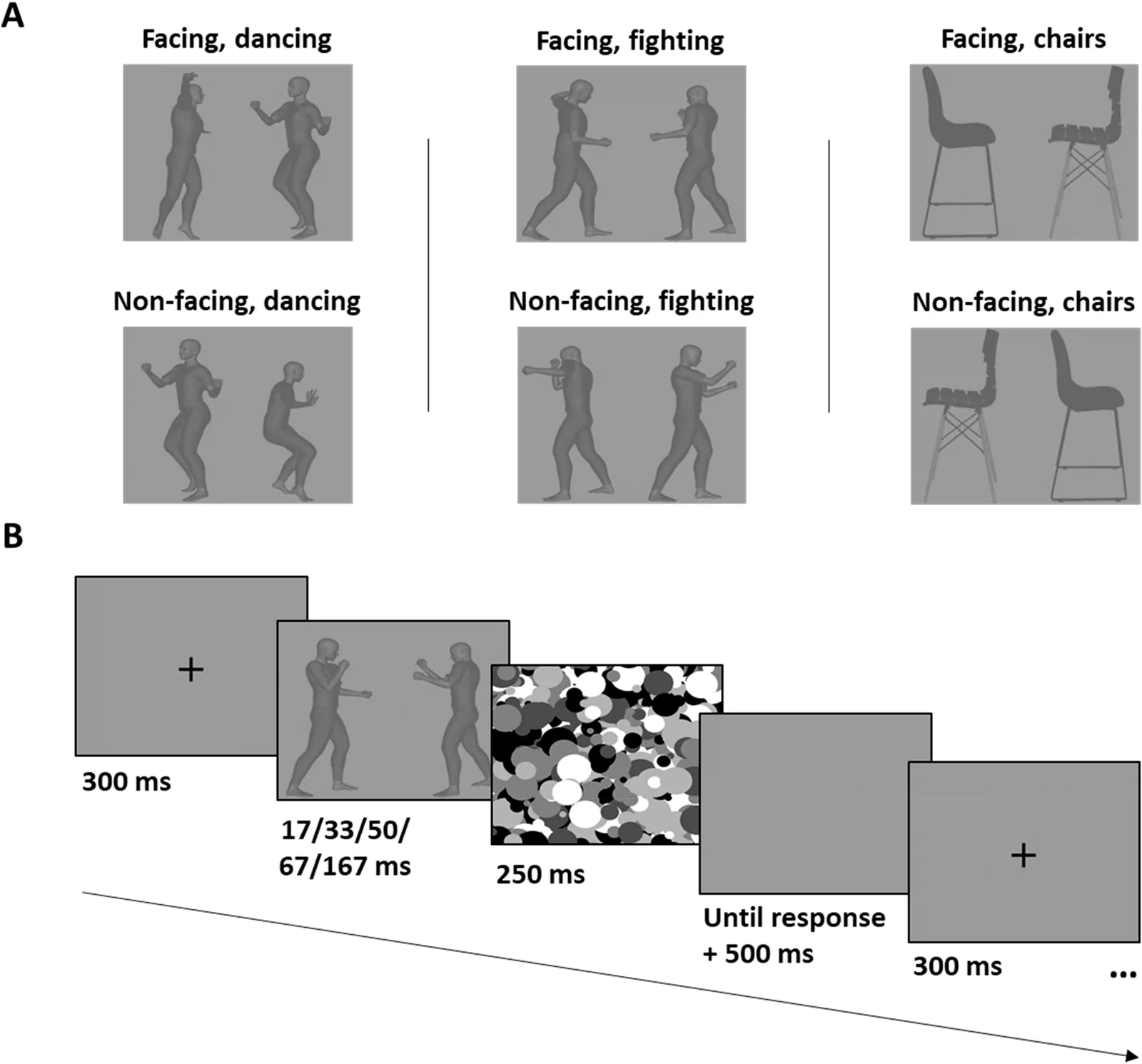
Examples of experimental stimuli (A) and design (B) of Experiment 1.

Stimuli were created from thirty grey-scale renderings of human bodies edited using Daz3D (Daz Productions, Salt Lake City, UT) and the Image Processing Toolbox of MATLAB (The MathWorks, Natick, MA). Individual body postures were compatible with one of two types of actions: fighting and dancing. To validate the experimenters’ selection of body postures, eleven participants (6 female, age range 21–25 years) external to the main study, were recruited online via Testable Minds, and asked to decide whether each individual body appeared to dance, fight, or perform another interaction. Body postures selected for the study achieved at least 70% agreement across participants, with mean agreement across postures of 89.51% ± 10.4 *SD* for dancing (range = 72.73%–100%) and 92.73% ± 11.17 *SD* for fighting (range = 72.73%–100%). Accordingly, ten “fighting” bodies and thirteen “dancing” bodies were selected. Two versions of each item were created by flipping the body along the vertical axis. Twenty dancing dyads and twenty fighting dyads were created by positioning two different bodies of the same category (i.e., dancing or fighting) face-to-face. By swapping the position of the two bodies in each dyad (the body on the left was moved to the right side of the image and the body on the right was moved to the left side of the image), we obtained the same number of ‘non-facing’ (i.e., back-to-back) dyads. Four additional dyads for each condition (facing and non-facing fighting and dancing) were created and used for training. The distance (number of pixels) between the closest points of two bodies in a dyad was matched between facing and non-facing dyads (*t*_(78)_ = 0.54, *p* = .589), facing and non-facing ‘fighting’ dyads (*t*_(38)_ = 0.74, *p* = .47), and facing and non-facing ‘dancing’ dyads (*t*_(38)_ = 0.24, *p* = .81). Dyads were resized to be shown at approximately 6° of visual angle, with ∼2° of visual angle between two bodies, from a distance of 60 cm. Bodies were shown on a light-grey background. Note that the very same stimulus-dyads were used in the three tasks tested in this study (object, relation, and interaction categorization). The same procedure as above was used to create twenty pairs of facing chairs and twenty pairs of non-facing chairs, starting from six different models of chairs. Pairs of chairs subtended a visual angle of 6° at a distance of 60 cm, with about ∼2° separating the two chairs. Chair stimuli were used in Task 1 (object categorization) and in a second version of Task 2, which therefore matched exactly the stimuli and conditions in Task 1 (see below). Masking stimuli were high-contrast Mondrian arrays (11° × 10°) of grey-scale circles (diameter = 0.4–1.8° of visual angle) (Figure 1A).

#### Procedure.

Before starting the experiment on Testable Minds, participants provided informed consent, and were instructed to set the room in a dim light, install the computer on a stable table, sit on a comfortable seat at about 60 cm (i.e., about an arm’s length) away from the screen, and turn off the sound of their computer and phone. Participants were randomly assigned to one of the following three tasks:

##### Task 1: Object Categorization.

Task 1 involved images featuring four types of stimuli, corresponding to four experimental conditions: facing body dyads (*n* = 20, 10 fighting and 10 dancing), and the corresponding non-facing body dyads (*n* = 20), facing chair pairs (*n* = 20), and the corresponding non-facing chair pairs (*n* = 20). Each stimulus was presented five times during the experiment, each time for a different duration: 17, 33, 50, 67, 167 ms (corresponding to 1, 2, 3, 4 and 10 frames of a 60-Hz refreshing rate-screen), yielding a total of 400 trials, presented in random order. Each trial involved the following sequence of events: fixation cross (300 ms), stimulus (17, 33, 50, 67 or 167 ms), mask (250 ms), and blank (a grey screen) until the participant’s response. The successive trial started 500 ms after the participant’s response (Figure 1B).

For each trial, participants were instructed to decide whether the stimulus depicted bodies or chairs. No other stimulus dimensions such as the action category (fighting/dancing) and positioning (facing/non-facing) were mentioned in the instructions. Participants had to respond as fast and accurately as possible, by pressing the keyboard keys “o” or “p”, with the right index and middle finger, respectively. The mapping between key and response (“bodies” or “chairs”) was counterbalance across participants. Accuracy and reaction times (RT) were recorded.

The experiment was preceded by two training phases with the same task instructions as in the actual experiment. In the first phase, eight stimuli (two for each condition) were presented for 250 ms, to familiarize the participants with the images. In the second phase, each of the eight stimuli (two for each condition) was presented five times, one for each of the five exposure durations, to familiarize the participants with the task. In both phases (but not in the actual experiment), feedback on the response accuracy was provided. The whole experimental session lasted ∼15 min.

##### Task 2: Relation Categorization.

Task 2 was identical to Task 1, except for the stimulus set, which only included body trials, and task instructions. Here, participants were asked to judge whether the two members of the dyad were oriented toward or away from each other. Participants had to respond, as fast and as accurately as possible, by pressing the keyboard keys “o” or “p” to respond “face-to-face” or “back-to-back”. The mapping between key and response (“face-to-face” or “back-to-back”) was counterbalance across participants. Since Task 2 only included body dyads, the total number of stimuli was doubled to match that of Task 1 (see the Supplementary Information for a different version of this task, replicating Task 2, but involving both bodies and chairs to entirely match stimuli and conditions of Task 1).

##### Task 3: Interaction Categorization.

Task 3 was identical to Task 2, except for the task instructions. Here, participants were asked to identify the type of interaction taking place between the two individuals in the dyad, that is, whether they appeared to fight or to dance. Participants had to respond, as fast and as accurately as possible, by pressing the keyboard keys “o” or “p” to respond “fighting” or “dancing”, respectively. The mapping between key and response (“fighting” or “dancing”) was counterbalance across participants.

#### Data Analysis.

We broke down the processing of minimal social interaction scenes into three processes: (i) categorization of the entities involved (bodies *vs.* chairs; Task 1); (ii) encoding of spatial relations (facing *vs.* non-facing; Task 2); and (iii) categorization of the interaction (fighting *vs.* dancing interactions; Task 3). We compared accuracy rates and RTs between the three tasks for stimuli presented for different durations, in order to measure task performance as a function stimulus duration, and thus investigate the temporal relationship between the three processes. Moreover, since the items in body-dyads were presented either face-to-face, a positioning that implies relationship or interaction (Papeo, 2020; Zhou et al., 2019), or back-to-back, we included the spatial relation as a factor, in order to examine whether and how the task performance changed depending on whether two items in the scene appeared to interact or not.

##### Preprocessing.

Data pre-processing and analysis were carried out in RStudio (R Core Team, 2024). Individual data points were inspected to detect and exclude trials with unrealistic response times, i.e., trials with RTs below 150 ms and above 3 seconds (1.43% of total trials) (see Stosic et al., 2024). In addition, we excluded trials in which participants’ screen refresh rate (recorded on a trial-by-trial basis) was slower than 60 Hz (single frame duration >17 ms), as this affected the exact stimulus duration (0.53% of total trials). In the remaining dataset, no participant had RTs above 3 standard deviations from the sample mean. For the RTs analysis, we only considered trials in which participants provided a correct response (82% of total trials).

##### Statistical Tests.

Accuracy data were modeled with a generalized linear mixed model, using the glmer function from the lme4 package (Bates et al., 2015) assuming a binomial distribution (Lee & Grimm, 2018). In doing so, we dealt with dependency in the data (within-subject factors). Our first model included three fixed effects—task (Object categorization, Relation categorization, Interaction categorization) as a between-subjects factor, stimulus duration (17, 33, 50, 67, 167 ms), and spatial relation (facing, non-facing) as within-subjects factors—and their interaction. By-subject intercepts were included as random effects. For the stimulus duration factor, Helmert contrast coding was used to assess uniformity of the differences between increasing ordered factor levels—each level was compared to the mean of subsequent levels (Brehm & Alday, 2022; Schad et al., 2020). Note that trials with chairs, which were only included in Task 1 to allow object categorization (i.e., bodies *vs.* chairs), were not considered in the main analysis (see Supplementary Information). Model fitting performance was evaluated with the r2 function from the performance package (Lüdecke et al., 2021), which returns adjusted *R*^2^ conditional (fixed + random effects contribution) and marginal (fixed effects contribution) values for generalized linear mixed models. The validity of model assumptions was evaluated using the DHARMa package (Hartig, 2024)—this procedure revealed that, for both main accuracy data models, model assumptions were met (Exp. 1: residuals distribution deviation test: *p* = 0.08; residuals over/under-dispersion test: *p* = 0.66; residuals outliers test: *p* = 0.35; Exp. 2: residuals distribution deviation test: *p* = 0.6; residuals over/under-dispersion test: *p* = 0.72; residuals outliers test: *p* = 0.69). To determine the statistical significance of our fixed effects predictors, we conducted a Type III Wald ANOVA using the car package (Fox & Weisberg, 2018). Standardized coefficients and their confidence intervals for generalized mixed models were calculated using the effectsize package (Ben-Shachar et al., 2020). Pairwise comparisons between critical conditions (*t*-tests) were run via the emmeans function from the lsmeans package (Lenth & Lenth, 2018; estimated marginal means given on the log odds ratio scale). Šidák correction was used to account for multiple comparisons (Abdi, 2007). Statistical threshold was set at *p* < .05. Plots were generated with the ggplot function from the ggplot2 package (Wickham, 2016). RTs data were modeled with a generalized linear mixed model approach assuming a gamma distribution (Lo & Andrews, 2015), with the same fixed and random effects factors as in the accuracy data analyses. Additionally, Bayes factors were computed as BF_10_ to assess evidence in favor of the alternative hypothesis (H_1_) over the null hypothesis (H_0_), using Bayesian generalized linear mixed models (with *brms*; Bürkner, 2017) and based on prior and posterior samples of a single parameter (using *bayestestR*; Makowski et al., 2019). This study was not preregistered.

### Results

**Figure 2.**
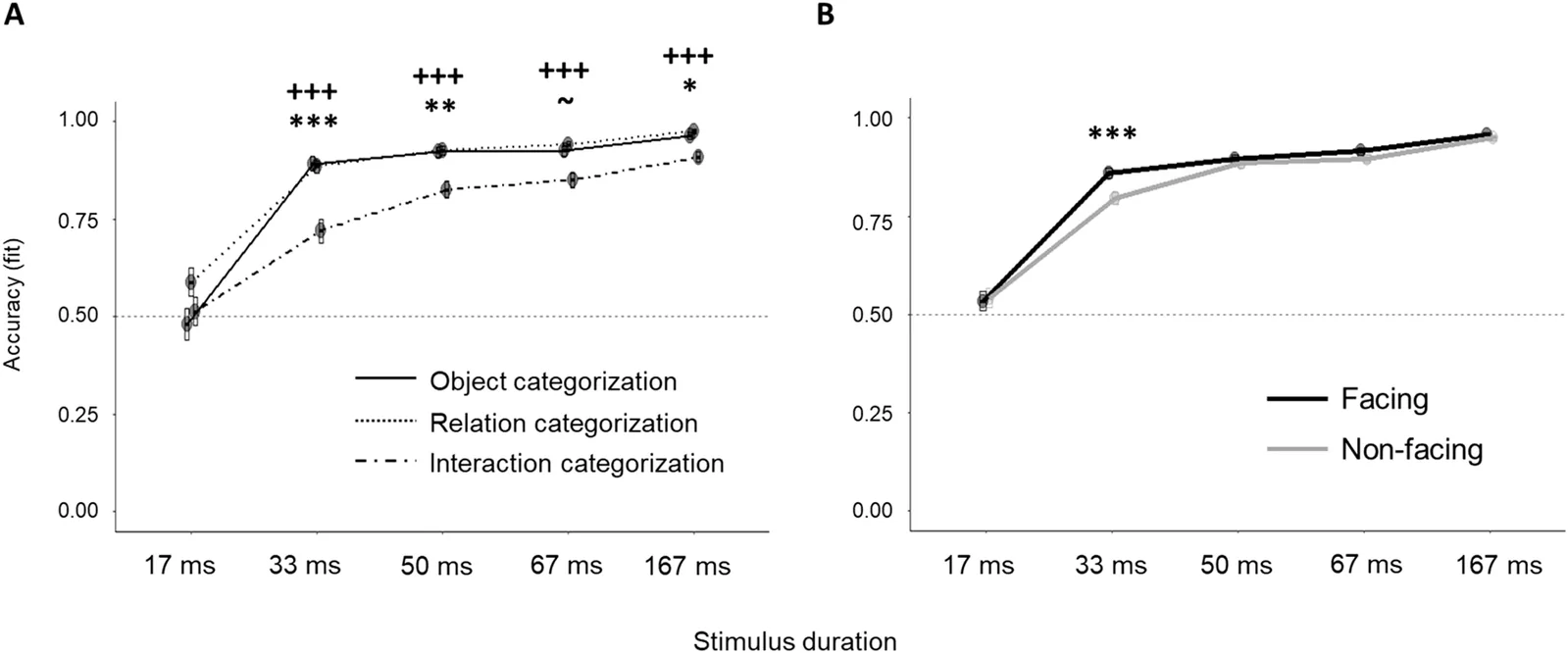
**Results of Experiment 1.** **A.** Interaction between Task and Stimulus duration revealing performance differences between the three tasks as a function of stimulus duration. Stars denote significant differences between Task 1 and 3; crosses denote significant differences between Task 2 and 3. **B.** Interaction between Spatial relation and Stimulus duration revealing an advantage for facing *vs.* non-facing dyads at 33 ms. ∼: *p* < .07; */+: *p* < .05; **/++: *p* < .01; ***/+++: *p* < .001. The horizontal dotted line indicates chance level performance.

As shown in Figure 2, in all tasks, performance became increasingly better as the presentation duration increased, with a pattern that was remarkably similar between Tasks 1 and 2, and differed between these two tasks and Task 3. Statistical analyses confirmed these observations. The statistical model fit to accuracy scores had goodness-of-fit of *R*^2^_conditional_ = .33 and *R*^2^_marginal_ = .24. We found a significant main effect of stimulus duration (*χ*^2^_(4)_ = 288.17, *p* < .001): accuracy increased significantly between 17 ms and all subsequent durations (*est.* = −2.43, *ste* = 0.14, *z* = −16.88, *p* < .001), between 33 ms and all subsequent durations (*est.* = −0.95, *ste* = 0.18, *z* = −5.22, *p* < .001), and between 67 ms and 167 ms (*est.* = −1, *ste* = 0.3, *z* = −3.37, *p* < .001). Note that this was also true for each individual task. In particular, accuracy at 33 ms was significantly lower than at 67, and 167 ms in Task 1, lower than performance at 50, 67 and 167 ms in Task 2, and lower than performance at 50, 67 and 167 in Task 3 (statistics for the comparisons between accuracy rates at 33 ms and other durations and between each pair of consecutive durations, for all tasks and experiments are reported in Supplementary Table S9). We also found a significant main effect of Task (*χ*^2^_(2)_ = 21.91, *p* < .001): accuracy was overall higher in Task 1 (*est.* = 0.69, *ste* = 0.21, *z* = 3.29, *p* = .003) and Task 2 (*est.* = 0.88, *ste* = 0.2, *z* = 4.33, *p* < .001) compared to Task 3, and did not differ between the first two tasks (*est.* = −0.2, *ste* = 0.22, *z* = −0.9, *p* = 0.74). Lastly, there were significant interactions between Task and Stimulus duration (*χ*^2^_(8)_ = 57.23, *p* < .001), and Stimulus duration and Spatial relation (*χ*^2^_(4)_ = 15.46, *p* = .004). No other main effects or interactions were significant (*p*s > 0.12; see Table S1 in Supplementary Information).

Pairwise comparisons addressing the significant Stimulus duration * Spatial relation interaction showed that the advantage of processing facing over non-facing dyads was significant at 33 ms of stimulus duration (*est.* = 0.51, *ste* = 0.1, *z* = 5.13, *p* < .001) (all other *p*s > 0.07) (Figure 2B).

Pairwise comparisons addressing the Task * Stimulus duration interaction revealed that accuracy rates for Task 1 and Task 2 did not differ from each other at any stimulus duration (all *p*s > .09 and all BF_10_s < 0.32, supporting the H_0_; a full report of statistics and corresponding BF_10_s for these comparisons is provided in Supplementary Information, Table S11), but were higher than in Task 3, at 33 ms (Task 1 *vs.* 3: *est.* = 1.13, *ste* = 0.24, *z* = 4.79, *p* < .001; Task 2 *vs.* 3: *est.* = 1.06, *ste* = 0.22, *z* = 4.84, *p* < .001), 50 ms (Task 1 *vs.* 3: *est.* = 0.85, *ste* = 0.25, *z* = 3.46, *p* = .008; Task 2 *vs.* 3: *est.* = 0.91, *ste* = 0.23, *z* = 4.01, *p* < .001), 67 ms (Task 1 *vs.* 3: *est.* = 0.71, *ste* = 0.25, *z* = 2.85, *p* = .06; Task 2 *vs.* 3: *est.* = 0.92, *ste* = 0.23, *z* = 3.98, *p* < .001), and 167 ms (Task 1 *vs.* 3: *est.* = 0.91, *ste* = 0.28, *z* = 3.25, *p* = .01; Task 2 *vs.* 3: *est.* = 1.26, *ste* = 0.26, *z* = 4.86, *p* < .001), but not at 17 ms (Task 1 *vs.* 3: *est.* = −0.15, *ste* = 0.22, *z* = −0.67, *p* = 1; Task 2 *vs.* 3: *est.* = 0.27, *ste* = 0.21, *z* = 1.31, *p* = .96) (Figure 2A). Put in another way, with 33 ms of exposure to the stimuli, performance improved dramatically (*vs.* 17 ms of exposure) in all three tasks, but significantly more in object and relation categorization (Tasks 1 and 2) relative to interaction categorization (Task 3). Note that these results were fully replicated when measuring performance in terms of *d primes* (*d*′), instead of accuracy rates (see Supplementary Information, p. 3; Figure S2).

We carried out two additional analyses. First, we performed analyses in the same way as above but excluding the 17-ms stimulus duration condition. In all three tasks, the performance at this presentation duration was dramatically worse than at other levels and, in effect, not different from chance (chance level = 0.5; Task 1: 0.48, *ste* = 0.04, *z* = −0.46, *p* = 1; Task 2: 0.59, *ste* = 0.04, *z* = 2.31, *p* = .27; Task 3: 0.51, *ste* = 0.04, *z* = 0.49, *p* = 1; Supplementary Table S2), and performance became more reliable, and therefore informative, from 33 ms of stimulus duration on. Results of the analysis without the level 17 ms of the stimulus duration variable confirmed all the effects reported above (Supplementary Information).

Second, in terms of structure, Task 1 differed more from the other two tasks as it was the only one that involved chair pairs—whereas the other two tasks only involved body dyads. Despite this, the performance pattern was remarkably similar between Task 1 and 2 (and different from Task 3). To take into account the structure of the task, we tested another group of participants with a different version of the relation categorization task (Task 2.1), in which facing and non-facing body dyads and chair pairs were presented, like in Task 1. In other words, Task 2.1 entirely matched Task 1 in terms of stimuli and conditions. A new analysis including data from Task 2.1 confirmed the above results (Supplementary Information, p. 4; Tables S3, S4; Figure S3). This analysis held that differences and similarities in performance across tasks captured the relationship between processes (i.e., object, relation, and interaction categorization) rather than differences and similarities in the superficial structure of the task (i.e., number of conditions and type of stimuli).

The key finding of Experiment 1 was that performance in Tasks 1 and 2 was similar across all stimulus durations, significantly better than in Task 3 and overall very accurate (Task 1: 87% ± 2.7 *SE*; Task 2: 87% ± 2.6 *SE*; Task 3: 72% ± 2.8 *SE*). However, if performance levels in Tasks 1 and 2 were too high—i.e., at ceiling—already at short stimulus duration (33 ms), this may have impacted the sensitivity of our test to detect differences between the two tasks. Statistical results allow this explanation to be ruled out. First, as reported above, performance in Tasks 1 and 2, as well as in Task 3, kept increasing as stimulus duration got longer (see Supplementary Table S9), meaning that it had not (yet) reached ceiling with 33-ms long stimuli. Second, Bayesian hypothesis testing via Bayes factors supported the absence of difference between Tasks 1 and 2.

RTs proved less sensitive than accuracy rates to the effects of experimental factors. The RT analysis showed an interaction between Task and Stimulus duration (*χ*^2^_(8)_ = 66.58, *p* < 0.001), reflecting differences across different levels of stimulus duration, for all three tasks, but no differences between the three tasks at any stimulus duration (the full report of RT results is provided as Supplementary Information; p. 2; Figure S1). Importantly, however, RT results did not contradict the accuracy results, thereby ruling out a speed/accuracy trade-off as an explanation of participants’ performance. This was further verified with additional analyses based on inverse efficiency scores (Bruyer & Brysbaert, 2011; Townsend & Ashby, 1983) and drift diffusion models (Ratcliff & McKoon, 2008), which combine accuracy and RTs into a single measure. The results of this analysis fully replicated those based on accuracy rates alone (see in Supplementary Information, pp. 18–20; Figures S12–S13).

## EXPERIMENT 2

Experiment 1 showed that recognition of bodies (body *vs.* chair categorization) and encoding of their spatial relation required similar processing times, as indicated by comparable levels of performance at each stimulus duration. Given the same stimulus duration, performance in interaction categorization was poorer than in the other two tasks, suggesting the need for additional processing times for the latter task. Experiment 2 was designed to replicate the results of Experiment 1, while also ensuring that participants’ performance reflected scene perception (the processing of the multiple items in the stimulus) rather than perception of single objects. In effect, in Experiment 1, all three tasks could be accomplished by either attending the two items, or just one. In particular, by only attending to the item on the left, one could successfully distinguish body *vs.* chair trials (Task 1), facing (body toward the center of the screen) *vs.* non-facing (body toward the periphery of the screen) trials (Task 2), and dancing vs. fighting trials (Task 3). Therefore, the three tasks in Experiment 2 were modified so that successful performance could only be achieved by processing both items in a stimulus.

### Materials and Methods

#### Participants.

Experiment 2 followed the same recruitment procedure as Experiment 1. A total of 64 participants were recruited and tested online in one of three tasks (Task 1: *n* = 22 participants, *M*_age_ = 27, *SD*_age_ = 5, 10 females; Task 2: *n* = 19 participants, *M*_age_ = 27, *SD*_age_ = 6, 13 females; Task 3: *n* = 23 participants, *M*_age_ = 27, *SD*_age_ = 5, 10 females). Sample size calculation for Experiment 2 drew upon Experiment 1. We run a sensitivity analysis on Experiment 1’s data by systematically decreasing the size of the interaction effect of interest—Task * Stimulus duration, in terms of *χ*^2^. This revealed that the smallest effect size of interest was *χ*^2^ = 20.52 (df = 8) (Supplementary Figure S4). Using this information, we carried out a power analysis using the pwr package in R (Champely et al., 2020). This revealed that with *α* = 0.05, and *β* = 0.8, the necessary sample size to detect an effect with *χ*^2^ = 20.52 was *N* = 48, while with a *β* = 0.9 it was *N* = 60 (Supplementary Figure S5).

#### Stimuli.

Stimuli in the three tasks were identical to Experiment 1. In addition, each task featured a new so-called ‘mixed’ condition. In Task 1, in addition to facing and non-facing bodies and chairs, we included ‘mixed’ pairs consisting of a body and a chair (20 facing and 20 corresponding non-facing pairs). In Task 2, in addition to facing and non-facing bodies, we included ‘face-to-back’ body dyads with the two bodies oriented in the same direction, or one facing the other who faced away (20 dyads oriented leftward and 20 dyads oriented rightward). In Task 3, in addition to facing and non-facing ‘dancing’ and ‘fighting’ dyads, we included ‘mixed’ dyads featuring a ‘dancing’ and a ‘fighting’ body (20 facing and 20 non-facing).

#### Procedure.

Procedures were identical to Experiment 1, except that we added a new ‘mixed’ condition in each task, and therefore the tasks had three response options. We reasoned that, if one only attended to one of the two items (e.g., the one on the left), performance in the ‘mixed’ conditions would be at chance. The ‘mixed’ conditions were therefore included to encourage participants to attend to the whole scene and to verify that they did so.

##### Task 1: Object Categorization.

In each trial, participants had to decide whether the stimuli depicted bodies, chairs, or a ‘mixed’ body-chair pair. If a participant only attended to one side of the screen (e.g., left), performance in the ‘mixed’ condition would be at chance. With the new condition, the task consisted of 600 trials—20 × 3 types of stimuli (bodies, chairs, mixed pairs) × 2 spatial positioning (facing dyads, non-facing dyads) × 5 exposure durations (17, 33, 50, 67, 167 ms). As in Experiment 1, participants had to respond, as fast and accurately as possible, by pressing the keyboard keys “o” or “p”, with the right index and middle fingers, for ‘bodies’ and ‘chairs’, respectively (the mapping between key and response was counterbalanced across participants). They were always instructed to respond using the key “e” with the left index for ‘mixed pair’.

##### Task 2: Relation Categorization.

Task 2 was identical to Task 1, except for the stimulus set, which included three types of body trials (facing, non-facing and ‘mixed’), and the task instructions. Participants had to judge whether the two members of the dyad looked toward or away from each other, or looked in the same direction (i.e., the ‘mixed’ condition). Keys “o” and “p” with the right index and middle fingers were used for ‘facing’ and ‘non-facing’ response, respectively (counterbalancing the key-response mapping across participants); key “e” with the left index was used for the ‘mixed’ response.

##### Task 3: Interaction Categorization.

Task 3 was identical to Task 2, except for the stimulus set and task instructions. Here, participants had to decide whether the two individuals in each dyad were fighting, dancing, or ‘mixed’ (i.e., one was in a ‘fighting’ posture and the other was in a ‘dancing’ posture). Keys “o” and “p” with the right index and middle fingers were used for ‘dancing’ and ‘fighting’ responses, respectively (counterbalancing the key-response mapping across participants); key “e” with the left index was used for the ‘mixed’ response.

#### Data Preprocessing and Analysis.

The same data preprocessing and analysis procedures as in Experiment 1 were implemented in Experiment 2. Like in Experiment 1, trials with RTs below 150 ms and above 3 seconds (3.9% of total trials) and trials in which the screen refresh rate was slower than 60 Hz (0.26% of total trials) were excluded. The mixed-pair conditions were not included in the analyses. This study was not preregistered.

### Results

**Figure 3.**
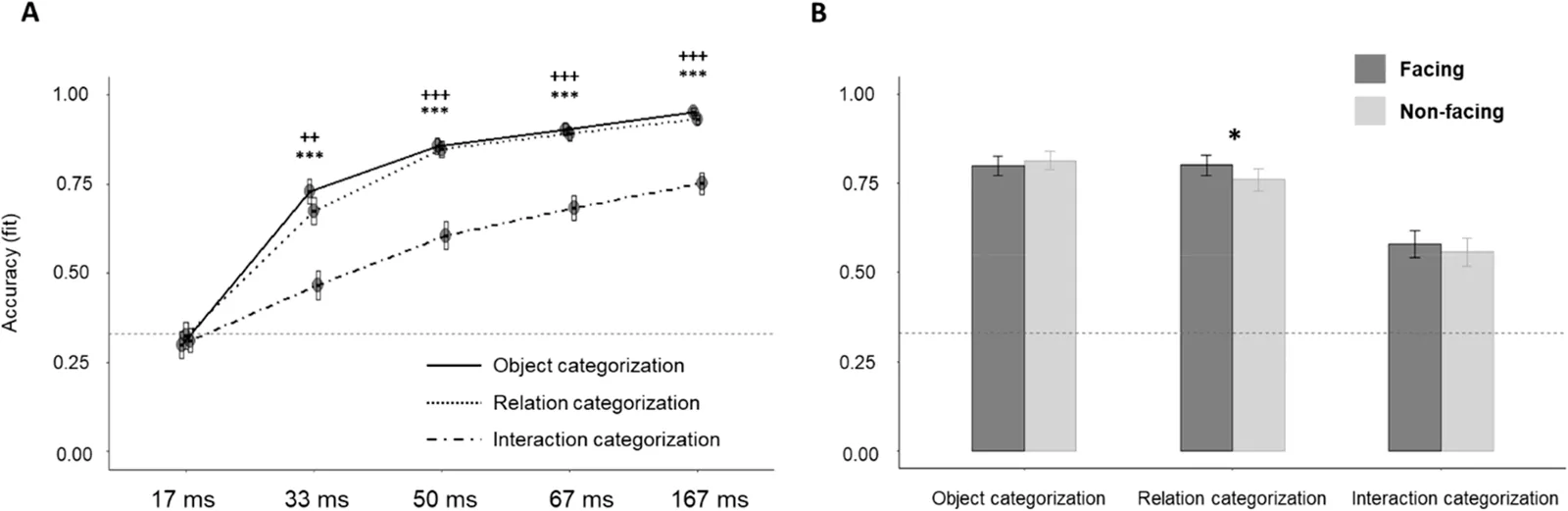
**A:** Performance for the three tasks in Experiment 2 as a function of presentation duration. Stars are related to the planned comparisons between Task 1 and Task 3; crosses are related to the planned comparisons between Task 2 and Task 3. **B:** Performance for facing vs. non-facing dyads at the three tasks. */+: *p* < .05; **/++: *p* < .01; ***/+++: *p* < .001. The horizontal dotted line indicates chance level performance (0.33).

As shown in Figure 3, Experiment 2 replicated the results of Experiment 1, showing an overall increase in performance accuracy with increasing stimulus duration, and a similar pattern of performance in Task 1 and Task 2, which differed from Task 3. Statistical analyses confirmed these observations. The model fit to the accuracy scores had goodness-of-fit of *R*^2^_conditional_ = .37 and *R*^2^_marginal_ = .26. Like Experiment 1, Experiment 2 revealed a significant effect of Stimulus duration (*χ*^2^_(4)_ = 467.13, *p* < .001), with an increase in accuracy rates from 17 ms to 167 ms of stimulus duration—differences were significant between 17 ms and all subsequent durations (*est.* = −3.13, *ste* = 0.15, *z* = −21.18, *p* < .001), 33 ms and all subsequent durations (*est.* = −1.45, *ste* = 0.16, *z* = −8.85, *p* < .001), 50 ms and all subsequent durations (*est.* = −0.82, *ste* = 0.2, *z* = −4.12, *p* < .001), and between 67 ms and 167 ms (*est.* = −0.75, *ste* = 0.27, *z* = −2.8, *p* = .005). The accuracy increase with increase in stimulus duration was true for each individual task. In particular, accuracy at 33 ms was significantly lower than at 50, 67, and 167 ms in Task 1, lower than performance at 50, 67 and 167 ms in Task 2, and lower than performance at 50, 67, and 167 in Task 3 (statistics for the comparisons between accuracy rates at 33 ms and other durations and between each pair of consecutive durations, for all tasks and experiments are reported in Supplementary Table S9). We also found a main effect of Task (*χ*^2^_(2)_ = 31.57, *p* < .001) reflecting higher accuracy in Task 1 (*est.* = 1.19, *ste* = 0.24, *z* = 5.03, *p* < .001) and Task 2 (*est.* = 1.02, *ste* = 0.24, *z* = 4.23, *p* < .001) compared to Task 3 and no difference between Tasks 1 and 2 (*est.* = 0.16, *ste* = 0.25, *z* = 0.65, *p* = .88). Finally, we found significant Task * Stimulus duration (*χ*^2^_(8)_ = 175.37, *p* < .001) and Task * Spatial relation interactions (*χ*^2^_(2)_ = 6.44, *p* = .03). No other main effects or interactions were significant (*p*s > .07; see Supplementary Tables S5, S6 for detailed statistics).

Pairwise comparisons investigating the Task * Stimulus duration interaction (*χ*^2^_(8)_ = 175.37, *p* < .001) revealed that accuracy rates in Task 1 and 2 did not differ at any stimulus duration (all *p*s > .99 and all BF_10_s < 0.9, supporting the H_0_; a full report of statistics and corresponding BF_10_s for these comparisons is provided in Supplementary Information, Table S11); but they both differed from Tasks 3 (Figure 3A). In particular, accuracy was higher in Task 1 than in Task 3, at 33 ms (*est.* = 1.06, *ste* = 0.25, *z* = 4.23, *p* < .001), 50 ms (*est.* = 1.49, *ste* = 0.26, *z* = 5.79, *p* < .001), 67 ms (*est.* = 1.57, *ste* = 0.26, *z* = 5.95, *p* < .001), and 167 ms (*est.* = 1.95, *ste* = 0.28, *z* = 6.97, *p* < .001), but not at 17 ms (*est.* = −0.13, *ste* = 0.25, *z* = −0.53, *p* = 1); likewise, accuracy was higher in Task 2 than in Task 3 at 33 ms (*est.* = 0.85, *ste* = 0.25, *z* = 3.41, *p* = .009), 50 ms (*est.* = 1.36, *ste* = 0.25, *z* = 5.35, *p* < .001), 67 ms (*est.* = 1.35, *ste* = 0.26, *z* = 5.2, *p* < .001), and 167 ms (*est.* = 1.51, *ste* = 0.27, *z* = 5.67, *p* < .001), but not at 17 ms (*est.* = 0.05, *ste* = 0.25, *p* = 1).

Pairwise comparisons investigating the Task * Spatial relation interaction (*χ*^2^_(2)_ = 6.44, *p* = .03) showed that accuracy was higher for facing than for non-facing dyads in the relation categorization task (*est.* = 0.19, *ste* = 0.07, *z* = 2.79, *p* = .01) (all other *p*s > 0.08) (Figure 3B). These results were fully replicated when measuring performance in terms of *d primes* (see Supplementary Information, p. 10; Figure S7).

An additional analysis showed that results did not change when removing trials with 17 ms of stimulus presentation, where performance was dramatically poorer than at other stimulus durations and, in effect, not different from chance (chance level = 0.33; Task 1: 0.28, *ste* = 0.04, *z* = −1.25, *p* = .97; Task 2: 0.32, *ste* = 0.04, *z* = −0.25, *p* = 1; Task 3: 0.31, *ste* = 0.03, *z* = −0.55, *p* = 1). Another analysis including data from Task 2.1 (a version of Task 2 including chair-stimuli) confirmed the above results (Supplementary Information, p. 12; Tables S7, S8; Figure S8).

Lastly, as in Experiment 1, reaction times (RTs) in Experiment 2 were less sensitive than accuracy rates to the effects of the experimental factors. Specifically, the RT analysis revealed an interaction between Task and Stimulus duration (*χ*^2^_(8)_ = 45.94, *p* < 0.001). This interaction indicated a difference between Task 1 and Task 3—RTs being faster in Task 1 for longer stimulus durations (50, 67, and 167 ms)—but no difference between Task 2 and Task 3. In addition, a difference emerged between Task 1 and Task 2.1, but not Task 2, at longer stimulus durations (for the full RT analysis, see Supplementary Information, pp. 10 and 13; Figures S6 and S8). The relatively low sensitivity of RTs to the experimental manipulations (i.e., differences between tasks), as well as their greater variability, may be attributable to the nature of the paradigm. In backward masking paradigms, stimulus perception is truncated, leading to processing failure (i.e., inaccurate responses) if processing is not completed before the onset of the mask (Enns & Di Lollo, 2000). This is particularly frequent at shorter exposure durations. Under these circumstances, RT analyses—which include only trials with correct responses—may be affected by imbalances across conditions when accuracy varies, as was the case here. It is also possible that RTs and accuracy reflect different aspects of performance, such as access to information (accuracy) vs. decision making (RTs). However, the fact that RT effects differed across Experiments 1 and 2 precludes any clear conclusion regarding what RTs captured in the present study. Importantly, however, RTs did not show a different pattern than accuracy—but just a noisier one—thereby ruling out a speed–accuracy trade-off (see also the analyses of inverse efficiency scores and drift diffusion models in the Supplementary Information, pp. 18–20).

## EXPERIMENT 3

Experiment 3 was designed to address the alternative explanation that performance in Task 3 differed from Tasks 1 and 2, not because of greater processing demands (and thus more processing failures under rapid stimulus presentation), but because of greater ambiguity at the level of stimuli. In particular, in Tasks 1 and 2, discriminations are in principle unambiguous (i.e., bodies *vs.* chairs; face-to-face *vs.* back-to-back stimuli). By contrast, some participants may have interpreted stimuli that we labeled as “fighting” as “dancing”, and *vice versa*—even though these labels were assigned to dyads composed of two bodies that were each consistently categorized as depicting either fighting or dancing postures (with ∼90% agreement; see Materials and Methods of Experiment 1). In this case, the lower accuracy rates in Task 3 would reflect a mismatch between our stimulus categorization and participants’ alternative—and equally valid—interpretations, rather than increased processing demands. To address this, in Experiment 3, we selected and included only unambiguous facing dyads as stimuli, that is, dyads that were consistently judged as either “fighting” or “dancing” by an independent panel.

### Materials and Methods

#### Participants.

A total of 67 participants were recruited and tested online in one of the three categorization tasks. Five of these participants displayed catastrophically low accuracy rates (<25%), possibly due to a misunderstanding of task instructions. Another participant had a screen refresh rate lower than 60 Hz. These six participants were excluded and replaced by six new participants, yielding a final sample size of 61 (Task 1: *n* = 20 participants, *M*_age_ = 35, *SD*_age_ = 7, 4 females; Task 2: *n* = 21 participants, *M*_age_ = 36, *SD*_age_ = 7, 7 females; Task 3: *n* = 20 participants, *M*_age_ = 35, *SD*_age_ = 7, 12 females).

#### Stimuli.

The stimuli for Experiment 3 were drawn from the set of Experiment 1, and were based on results of a new online rating study, in which dyad stimuli rather than single body postures, were rated for interaction content. In this study, a panel of 20 participants, independent to the main experiment, judged whether each facing dyad involved in the original set (20 labeled as “fighting” and 20 as “dancing”) depicted a fight, a dance, or another interaction. We found that 9 “fighting dyads” out of the original 20 were interpreted as “fight” interactions with 100% of agreement across participants (20/20 raters), 8 dyads obtained an agreement of 95%, and 3 reached 90%. For the “dancing set”, one dyad obtained 100% of agreement on “dance”, 4 obtained 95% of agreement (19/20 raters), 13 obtained an agreement of ≥70%, and 2 dyads reached an agreement of ≥60%. We report in Supplementary Information (p. 23) more details on this study. We selected the 9 fighting dyads with 100% agreement and the 5 dancing dyads with 95–100% agreement. Experiment 3 involved the selected set and the corresponding mirror-flipped versions, yielding 18 fighting and 10 dancing dyads with high inter-rater agreement.

#### Procedure.

Procedures were identical to Experiment 1, except that Task 1 (object categorization) and Task 3 (interaction categorization) included only the above selected facing dyads; in Task 2 (relation categorization) in addition to the selected facing set, we presented the corresponding non-facing dyads, to allow the relation-categorization task (face-to-face vs. back-to-back categorization). As in the original studies, each task consisted of 400 trials. To reach this number, tasks included a higher repetition of the same stimuli (Tasks 1 and 2: five to six repetitions for fighting dyads, ten repetitions for dancing dyads; Task 3: eleven to twelve repetitions for fighting dyads, twenty repetitions for dancing dyads).

#### Data Preprocessing and Analysis.

The same data preprocessing and analysis procedures as in Experiments 1 and 2 were implemented in Experiment 3. Trials with RTs below 150 ms and above 3 seconds (1.2% of total trials) and trials in which the screen refresh rate was slower than 60 Hz (0.6% of total remaining trials) were excluded. This study was not preregistered.

### Results

**Figure 4.**
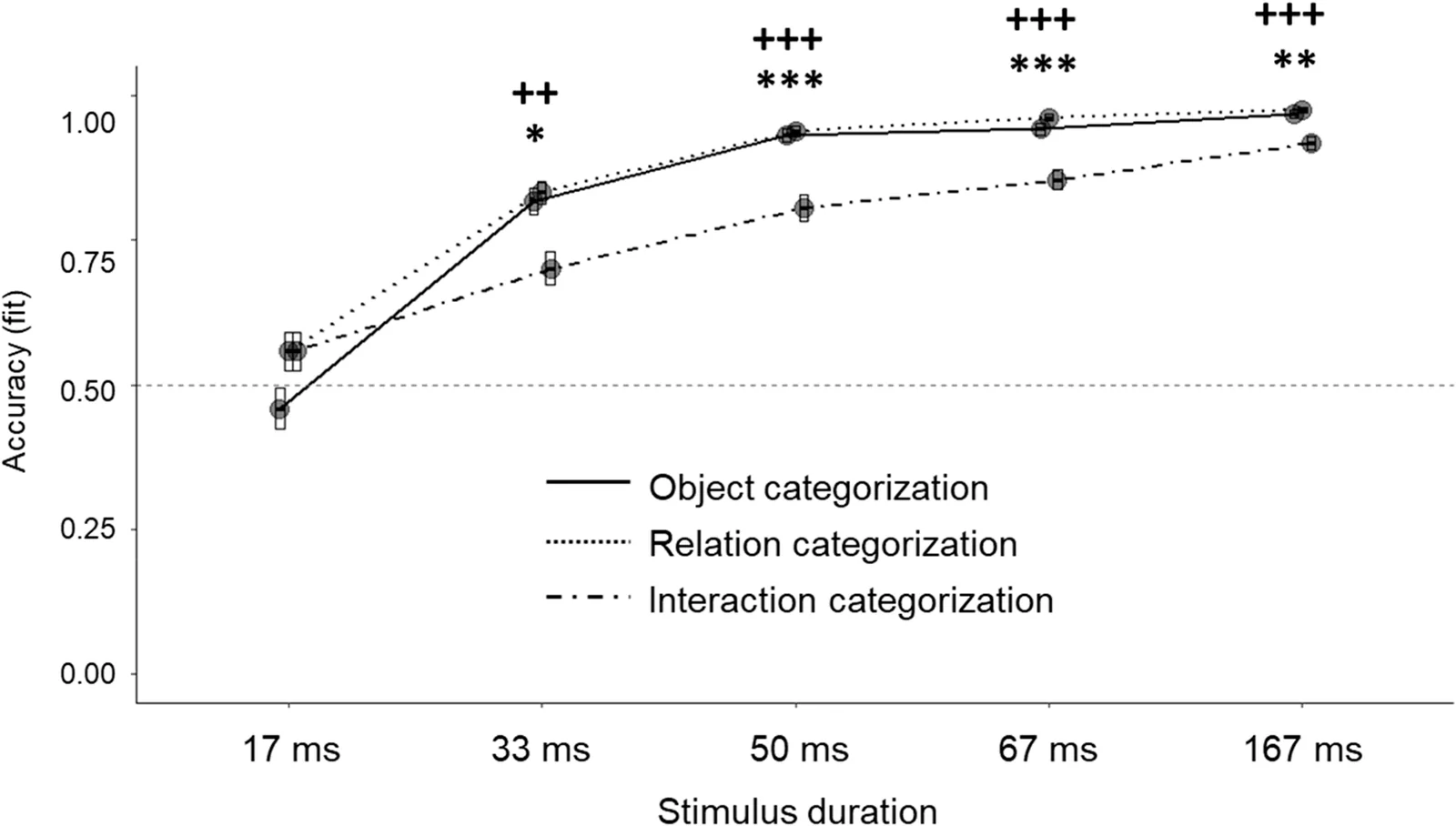
**Results of Experiment 3.** Interaction between Task and Stimulus duration revealing performance differences between the three tasks as a function of stimulus duration. Stars denote significant differences between Tasks 1 and 3; crosses denote significant differences between Tasks 2 and 3. */+: *p* < .05; **/++: *p* < .01; ***/+++: *p* < .001. The horizontal dotted line indicates chance level performance.

As shown in Figure 4, Experiment 3 replicated the results of Experiments 1 and 2, showing comparable performance in Tasks 1 and 2 across all stimulus-presentation durations, and lower accuracy rates in Task 3 across all presentation durations (except 17 ms, where performance was a chance like as previous in previous experiments). This was confirmed by statistical analyses. The model fit to the accuracy scores had goodness-of-fit of *R*^2^_conditional_ = .33 and *R*^2^_marginal_ = .27. We found a significant effect of Stimulus duration (*χ*^2^_(4)_ = 688.57, *p* < .001), with an increase in accuracy rates from 17 ms to 167 ms of stimulus duration—differences were significant between 17 ms and all subsequent durations (*est.* = −2.74, *ste* = 0.1, *z* = −25.97, *p* < .001), 33 ms and all subsequent durations (*est.* = −1.42, *ste* = 0.13, *z* = −11.1, *p* < .001), 50 ms and all subsequent durations (*est.* = −0.49, *ste* = 0.18, *z* = −2.78, *p* < .001), and between 67 ms and 167 ms (*est.* = −0.61, *ste* = 0.23, *z* = −2.78, *p* = .009). We also found a main effect of Task (*χ*^2^_(2)_ = 29.27, *p* < .001) reflecting higher accuracy in Task 1 (*est.* = 0.65, *ste* = 0.19, *z* = 3.66, *p* < .001) and Task 2 (*est.* = 0.95, *ste* = 0.18, *z* = 5.25, *p* < .001) compared to Task 3 and no difference between Tasks 1 and 2 (*est.* = −0.27, *ste* = 0.19, *z* = −1.41, *p* = .4). Finally, we found a significant Task * Stimulus duration interaction (*χ*^2^_(8)_ = 210.69, *p* < .001).

Pairwise comparisons investigating this interaction revealed that accuracy rates in Task 1 and 2 did not differ at any stimulus duration (all *p*s > .45); but they both differed from Task 3. In particular, accuracy was higher in Task 1 than in Task 3, at 33 ms (*est.* = 0.65, *ste* = 0.2, *z* = 3.15, *p* = .02), 50 ms (*est.* = 1.17, *ste* = 0.23, *z* = 5.13, *p* < .001), 67 ms (*est.* = 1.01, *ste* = 0.24, *z* = 4.26, *p* < .001), and 167 ms (*est.* = 0.99, *ste* = 0.27, *z* = 3.67, *p* = .003), but not at 17 ms (*est.* = −0.4, *ste* = 0.2, *z* = −2.05, *p* = .46); likewise, accuracy was higher in Task 2 than in Task 3 at 33 ms (*est.* = 0.75, *ste* = 0.25, *z* = 3.87, *p* = .001), 50 ms (*est.* = 1.3, *ste* = 0.21, *z* = 6.23, *p* < .001), 67 ms (*est.* = 1.46, *ste* = 0.22, *z* = 6.5, *p* < .001), and 167 ms (*est.* = 1.25, *ste* = 0.24, *z* = 5.11, *p* < .001), but not at 17 ms (*est.* = −0.004, *ste* = 0.10, *z* = −0.02, *p* = 1) (Figure 4).

Furthermore, we repeated these analyses on the selected fighting dyads only, for which the inter-rater agreement was 100%. The results showed no significant departure from previous findings. The full report of these additional analyses is provided in Supplementary Information (p. 25).

As in the previous two experiments, RTs in Experiment 3 were less sensitive than accuracy to our experimental manipulation. This analysis revealed an interaction between Task and Stimulus duration (*χ*^2^_(8)_ = 33.8, *p* < .001). This interaction indicated a difference between Task 1 and Task 3—RTs being faster in Task 1 (at 17, 33, 50, and 67)—but no difference between Task 2 and Task 3. In addition, a difference emerged between Task 1 and Task 2 at all stimulus durations (for the full RT analysis of Experiment 3, see Supplementary Information, pp. 24–25; Figure S14).

Experiments 3 constitutes a fourth replication of the same pattern of results, observed across: the original stimulus set (Experiment 1); the analysis including a modified set (Task 2.1) to increase stimulus similarity between Tasks 1 and 2 (Experiments 1–2; Supplementary Information); a set introducing a “mixed” condition for each task (Experiment 2); and, here, a set only comprising semantically unambiguous interactions. Together, these converging findings indicate that the observed differences and similarities between tasks reflect underlying processing differences and commonalities, rather than idiosyncrasies of the specific stimuli used in each task.

## DISCUSSION

What are the processing steps involved in forming a mental representation of a social interaction? Here, we addressed this question by considering the relationship between three main computations required for social interaction recognition: recognition of social agents (*vs.* other entities), encoding of their spatial relations and of their actions. We systematically manipulated the stimulus duration, therefore the time allowed for each of the three tasks, reasoning that comparable levels of performance for a given stimulus duration in different tasks would indicate comparable processing times (de la Rosa et al., 2011; Grill-Spector & Kanwisher, 2005).

Three experiments provided remarkably consistent results. First, the recognition of social agents (i.e., people) and of their spatial relation (face-to-face *vs.* back-to-back) yielded comparable performance levels at every stimulus duration, from 17 ms to 167 ms. Second, performance at every stimulus duration was consistently less accurate for interaction categorization, suggesting that this task required longer processing times than the other two. Third, in the first two tasks, performance with 33-ms stimulus duration improved dramatically relative to the condition with 17-ms stimulus duration, and kept improving at increasing stimulus durations, but with a gain that was visibly smaller than in the interaction categorization task. Moreover, in this last task, performance with 33-ms stimulus duration improved relative to the condition with 17-ms stimulus duration, but significantly less that in the other two tasks, and it kept improving at increasing stimulus durations, with a gain that was visibly larger than in the other two tasks. In other terms, performance in interaction categorization benefited from additional processing time more than performance in the other tasks. Finally, in both experiments, task performance showed an overall advantage for processing facing people, over non-facing people.

Thus, a key finding of Experiments 1, 2 and 3 was that performance in object categorization (Tasks 1) and relation categorization (Task 2) was similar across all stimulus durations, and significantly different—better—than in Task 3. Before discussing the implications of these findings, we consider—and rule out—an alternative explanation based on task difficulty. If performance levels were too high and reached ceiling already at 33 ms of stimulus duration, in Tasks 1 and 2, but not in Task 3, this may have impacted the sensitivity of our test to detect differences between the first two tasks. Statistics however speak against this interpretation. First, Bayesian hypothesis testing provided statistical evidence in favor of the absence of difference between Tasks 1 and 2. Second, and more critically, it is true that performance in Tasks 1 and 2 with 33-ms stimulus duration was quite high in Experiment 1 (87% in both tasks). However, in Experiment 2, accuracy dropped to 70% and 66% in Tasks 1 and 2, respectively. Thus, results of Experiment 2, which fully replicated the effects of Experiment 1 with more difficult tasks (tasks were made more difficult by including a third response option), mitigate the concern that absence of differences between Tasks 1 and 2 was merely due to a ceiling effect. Finally, in both experiments, accuracy at 33 ms of stimulus duration in Tasks 1 and 2 did not meet the criteria for a ceiling effect. A celling effect occurs when increases in the independent variable (here, stimulus duration) no longer produce changes in the dependent variable (here, accuracy) (Taylor, 2010). In contrast, in both Experiments 1 and 2, and in both Tasks 1 and 2, accuracy increased significantly with longer stimulus durations (see Results and Supplementary Information, Tables S9 and S10), indicating that performance had not yet reached ceiling at 33 ms of stimulus duration. Taken together, these findings indicate that performance in Tasks 1 and 2 was not at ceiling, and that our paradigm was sufficiently sensitive to detect differences between tasks, when differences existed.

### Object Recognition and Relation Perception

A corpus of studies has shown that visuo-spatial information relevant for human social interaction (e.g., interpersonal orientation and distance, postural relations) is extracted automatically upon stimulus presentation, in paradigms that emphasize perceptual processing of the stimuli, as opposed to slower inferential processes (Fu et al., 2024; Goupil et al., 2025; Papeo et al., 2017, 2019; Su et al., 2016). This literature has initially suggested that, between the perception of social entities and the recognition of social interactions, there is a stage for ‘relation perception’, that is, for encoding relational features that specify whether and how two social agents are related (Hafri & Firestone, 2021; Malik & Isik, 2023; McMahon & Isik, 2023; Papeo, 2020). However, this literature has not addressed the relationship between recognition of objects, actions and relations. The present study represents a first and novel advance in this field: By showing that processing times are comparable for categorization of objects and of their relations, it suggests that relation perception occurs *together with* object recognition.

Given the parallel between the two processes, one might wonder whether they are mediated by the same perceptual mechanism, or relation perception is even part of object recognition. There is evidence that meaningful spatial relations between objects aid object recognition. For example, a body in a spatial configuration that implies relationship/interaction with a nearby body is recognized more easily and represented better in visual cortex than the same body presented in isolation (Abassi & Papeo, 2020; Ding et al., 2017; Papeo et al., 2017; Paparella & Papeo, 2022; Xu et al., 2023; see also Kaiser et al., 2014, 2019). Moreover, evidence suggests that both the processing of social entities and of their relations rely on the lateral occipitotemporal cortex (LOC) (Abassi & Papeo, 2020, 2022, 2024; Bellot et al., 2021; Landsiedel et al., 2022; McMahon & Isik, 2023; Walbrin & Koldewyn, 2019; Wurm & Caramazza, 2019), and that in this brain area, neural activity patterns associated with perception of spatial relations correlate with those associated with perception of the agents involved in the relations (Dima et al., 2022).

Even so, it remains an open question whether the two processes are independent. Relations may be visual properties that help define an object representation (similarly to visual features such as shape and texture), such that a failure in relation perception could perturb object recognition. Alternatively, relations could be represented independently from the objects within the relation, so that one could see, say, the “facingness” between two objects without accessing the objects’ categories or identities (see for discussion, Hochmann & Papeo, 2021). These questions, critical for developing models of human recognition of social interaction, should be addressed in future *ad hoc* experiments, for instance, to demonstrate that if one process fails the other fails too, or that the encoding of object categories and of object relations have different neural implementation (see Abassi & Papeo, 2024, for related fMRI investigation).

### The Advantage of *Social Facingness*

Our results carry several indications for an overall advantage in processing facing, over non-facing bodies. Performance with facing bodies was qualitatively better than performance with non-facing bodies in all tasks and at all stimulus durations (from 33 ms onwards) in Experiment 1 (with a significant effect at a stimulus duration of 33 ms), and in the relation and interaction categorization tasks (with a significant effect in the interaction categorization) in Experiment 2. Further analyses showed that the advantage of *facingness* was specific for the social condition, i.e., the condition involving bodies (see “Comparisons between bodies and chairs” in Supplementary Information, p. 14). In particular, statistically significant interactions in tasks that involved both body- and chair-trials (Task 1 and Task 2.1) showed that accuracy was higher with facing (*vs.* non-facing) dyads, for bodies, but not for chairs. Moreover, performance was significantly higher in the interaction categorization task (i.e., deciding on “fighting” *vs.* “dancing”) when stimuli presented body dyads face-to-face (i.e., main effect of Spatial relation in “Exploring the effect of spatial relation on interaction categorization” of Supplementary Information, p. 17). This finding confirms that *facingness* is a key cue for the recognition of social interactions.

The processing advantage of *social facingness* is an empirical phenomenon, replicated in many studies using various types of stimuli and tasks. This corpus of studies has shown that two facing bodies are more likely to be detected and recognized than the same bodies facing away from each other (Gandolfo et al., 2024; Goupil et al., 2025; Papeo et al., 2017; Papeo & Abassi, 2019), with effects that impact the very early, pre-attentive stages of visual perception (Fu et al., 2024; Papeo et al., 2019; Su et al., 2016; Xu et al., 2023), up to memory (Ding et al., 2017; Lu et al., 2022; Paparella & Papeo, 2022; Vestner et al., 2019; Wu et al., 2024). Along with those studies, here, the advantage for social facingness emerged rapidly, i.e., with 33 ms of stimulus duration, and tended to be particularly strong just at this delay, suggesting that this behavioral phenomenon singles out the early, visual perception stages of social interaction processing (Gandolfo et al., 2024). Moreover, like many effects emerging in visual perception (Hafri & Firestone, 2021), the advantage of social facingness appears to be automatic, as here it was found in a task that explicitly required processing relations as well as in tasks where this aspect of the stimuli was left implicit.

The social facingness effect is important in that it shows that relation perception is a sophisticated process, which not only discriminates visual scenes based on relational information, but also uses that information to give priority to stimuli with social relevance, such as two facing bodies (Papeo, 2020; Papeo et al., 2026). As discussed above, our results leave open the possibility that relations are represented independently from the objects involved in the relation. However, even if so, the facingness effect with short stimulus durations implies that information about object categories and relations is rapidly integrated, yielding an advantage in processing facing *vs.* non-facing bodies—but not facing *vs.* non-facing chairs.

### Categorization of Social Interactions

Participants required a stimulus presentation of 167 ms for interaction categorization to reach the same performance level that, in the other two tasks, was achieved with 33 ms of stimulus duration. That is, interaction categorization required longer processing time than the other two tasks.

Before discussing the implications of these findings, we consider—and rule out—an alternative explanation based on task semantic ambiguity. In particular, in Experiment 3, we tested whether poorer performance in Task 3 reflected a mismatch between the experimenters’ labels and the participants’ interpretations of the stimuli as “fight” or “dance”, rather than greater processing demands. In particular, while Tasks 1 and 2 involved unambiguous discriminations (i.e., bodies vs. chairs; face-to-face vs. back-to-back stimuli), stimuli in Task 3 could be interpreted differently (some may have interpreted stimuli that we labeled as “fighting” as “dancing”, or *vice versa*). Experiment 3 replicated the results of Experiments 1 and 2 using highly unambiguous stimuli (i.e., dyads classified as “fight” or “dance” events by the 100% or the 95% of an independent panel). This supports the interpretation that lower accuracy rates in Task 3 reflects greater processing demands (and thus more processing failures under rapid stimulus presentation, relative to Tasks 1 and 2), not variations in stimulus interpretation.

The extra time needed for interaction categorization may imply the recruitment of different and/or additional mechanisms. The former interpretation is compatible with neuroimaging results showing that categorical distinctions between social interactions, such as helping *vs.* hindering, or joint action *vs.* communication, are encoded in regions upstream of those representing relational (e.g., facingness) information. In particular, social-interaction categorization has been consistently associated with activity in the superior temporal cortex, which lays anteriorly and superiorly to LOC (Isik et al., 2017; Lee Masson & Isik, 2021; McMahon et al., 2023; Walbrin et al., 2018) and, in a hierarchical model of social interaction processing, receives projections from LOC regions, and represents social interactions at a more abstract level than faces, bodies (i.e., social entities) and their relations (McMahon & Isik, 2023; Pitcher & Ungerleider, 2021).

The extra time needed for interaction categorization may also reflect the requirement for finer-grained perceptual distinctions. In this interpretation, deciding on ‘fighting’ *vs.* ‘dancing’ involves discriminating between postural cues that may be subtler than the coarse distinctions to discriminate between bodies and chairs (Task 1) or facing and non-facing (Task 2). Besides differences in perceptual difficulty, tasks also differed in terms of categorization level—e.g., basic for the bodies *vs.* chairs and subordinate for fighting *vs.* dancing. In this respect, it remains possible that individuals are capable of detecting social interactions (e.g., reporting whether two interact or not) before discriminating between specific interaction categories (e.g., fighting *vs.* dancing). Hypothetically, when individuals discriminate between facing and non-facing people, they could also represent the distinction between interacting and non-interacting, such that the two processes would overlap in terms of processing times. A magnetoencephalography study (Isik et al., 2020) showed that detection of social interaction (i.e., the mere discrimination between interacting and non-interacting people) occurs relatively late (i.e., from 300 ms). However, no study has so far compared the time course to encode relations (facing *vs.* non-facing), judge whether two interact or not, and access the interaction category (fighting *vs.* dancing).

The present study investigated whether and how three tasks performed on identical stimuli differ in their processing time. Elucidating the specific mechanisms underlying these differences remains an important goal for future studies.

## CONSTRAINTS ON GENERALITY

The findings and interpretations of this work can be safely generalized to the population of young adults with no diagnosis of psychiatric or neurological conditions, for participants were recruited via an online recruiting platform (Testable Minds) that ensured the heterogeneity of participants’ demographic background. Moreover, although we do not expect cultural differences, these results should be replicated in populations living outside Europe or North America, where all our participants were located. Lastly, this work’s findings might not generalize to individuals with psychiatric and/or neurological conditions that impact social perception and social cognition (Derntl & Habel, 2011).

## CONCLUSIONS

This study addressed the relationship, in terms of processing times, between three main processing steps for visual recognition of social interactions: recognition of social (*vs.* non-social) entities, encoding of their spatial relations and interaction categorization. Results showed that it takes no longer to access the relation between objects in a scene (facing *vs.* non-facing) than to access the objects’ category (bodies *vs.* chairs), while substantially more processing is required in order to categorize social interactions (fighting *vs.* dancing). The striking parallel between object and relation categorization demonstrates that relations between objects are extracted as rapidly as categorical object information, providing the building blocks for social interaction recognition. These findings impose important constraints, and provide useful indications for current attempts to model human recognition of social interactions.

## FUNDING INFORMATION

Funding for this project was provided by a European Research Council Starting Grant awarded to L. P. (Project: THEMPO, Grant Agreement 758473). M. M. was supported by a fellowship of the LabEx CORTEX of the University of Lyon (ANR-11-LABX-0042).

## AUTHOR CONTRIBUTIONS

M.M.: Conceptualization; Data curation; Formal analysis; Methodology; Visualization; Writing – original draft; Writing – review & editing. J.D.: Conceptualization; Data curation; Formal analysis; Investigation; Methodology; Writing – original draft. C.S.: Data curation; Formal analysis. L.P.: Conceptualization; Funding acquisition; Methodology; Supervision; Writing – original draft; Writing – review & editing.

## DATA AVAILABILITY STATEMENT

We reported all data pre-processing and analysis steps, and the software used to carry them out, in the Data analysis sections. Data and code are publicly available at: https://osf.io/mfv39/overview?view_only=886ffc071fd54f0f9f43946d3a59b263.
